# Do Dairy Farming Systems Differ in Antimicrobial Use?

**DOI:** 10.3390/ani10010047

**Published:** 2019-12-25

**Authors:** Anna Zuliani, Isabella Lora, Marta Brščić, Andrea Rossi, Edi Piasentier, Flaviana Gottardo, Barbara Contiero, Stefano Bovolenta

**Affiliations:** 1 Department of Agricultural, Food, Environmental and Animal Sciences, University of Udine, 33100 Udine, Italy; zuliani.anna.2@spes.uniud.it (A.Z.); andrearossi1992@gmail.com (A.R.); edi.piasentier@uniud.it (E.P.); stefano.bovolenta@uniud.it (S.B.); 2 Department of Animal Medicine, Production and Health, University of Padua, 35020 Padua, Italy; flaviana.gottardo@unipd.it (F.G.); barbara.contiero@unipd.it (B.C.)

**Keywords:** treatment incidence, CIA, dairy cattle, intensive farming, mountain farming, high-yielding breeds, dual-purpose breeds

## Abstract

**Simple Summary:**

The overuse and misuse of antimicrobials in dairy farming may lead to the development of antimicrobial resistance and thus to the reduction of the antimicrobial treatment efficacy against animal or human bacterial diseases. This study aims to investigate antimicrobial use differences in four farm groups: mountain farms with specialized high-yield dairy breeds or with dual-purpose breeds raised for milk production, and lowland farms with specialized high-yield dairy breeds or with dual-purpose breeds raised for milk production. From the results, we found a significant difference between mountain farms with dual-purpose breeds and lowland farms with specialized breeds for the overall antimicrobial use and for the use of those antimicrobial classes that are most important in human medicine. Mountain farms have a generally lower milk production and smaller herd size than lowland farms, provide cows with access to pasture, and limit concentrates in the diet. These management practices and the use of local/dual-purpose breeds could reduce the risk of production diseases and the consequent need for antimicrobial use.

**Abstract:**

The quantitative assessment of antimicrobial use (AMU) in food-producing animals contributes to the provision of essential information for developing relevant and effective policies to reduce use and to control antimicrobial resistance. Information on AMU is available mainly for intensive dairy farming systems and specialized high-yielding breeds. The aim of this study is to investigate AMU in different dairy farming systems by comparing the treatment incidence in mountain farms with specialized high-yield dairy breeds or with dual-purpose breeds raised for milk production to the treatment incidence in lowland farms with specialized high-yield dairy breeds or with dual-purpose breeds raised for milk production. Significant differences were found only between the overall treatment incidence, as well as the treatment incidence of highest-priority critically important antimicrobials for human medicine, in lowland farms with high-yielding breeds and mountain farms with dual-purpose breeds. Mountain farms have a generally lower milk production and smaller herd size than lowland farms, provide cows with access to pasture, and limit concentrates in the diet. These management practices and the use of local/dual-purpose breeds could reduce the risk of production diseases and the consequent need for AMU.

## 1. Introduction

Antimicrobial resistance (AMR) is considered a global threat across the human and animal health sectors. The overuse and misuse of antimicrobials in human and veterinary medicine is the leading cause of the emergence of antimicrobial resistance in bacteria. In order to control the phenomenon, the World Health Organization (WHO) produces and regularly updates a classification and prioritization list of antimicrobials according to their importance for human medicine. The list identifies three main categories: Critically important antimicrobials (CIA), highly important antimicrobials (HIA), and important antimicrobials [1]. Within CIA, two subcategories are identified: Highest-priority CIA (HP-CIA), which includes cephalosporins of the 3rd, 4th, and 5th generation, glycopeptides, macrolides, ketolides, polymyxins, and quinolones; and high-priority CIA (H-CIA). Resistance against these substances can dramatically limit the treatment options against serious human bacterial diseases and should thus be prudently used in veterinary medicine. Despite the still-unclear livestock–human AMR transfer route, there is evidence of the strong interaction between antimicrobial use (AMU) and AMR in the livestock sector [2].

The quantitative assessment of AMU in food-producing animals contributes to the provision of essential information for developing relevant and effective policies to reduce AMU and to control AMR.

In the dairy sector, several studies were carried out in an attempt to compare metrics [3,4], quantify consumption [5,6,7], or investigate associations between AMU and management practices or farm performance [8,9].

Italy is one of the greatest users of antimicrobials for food-producing animals in Europe [10], but little information is available on AMU in dairy cattle and, when available, it focuses on intensive farming systems and high-yielding breeds [11].

In general terms, farming systems may differ in herd size, breed type, use of pasture, husbandry system, and dietary inputs provided to cows. Traditional mountain farming systems are known to be smaller in herd size, adopting traditional management practices and local/dual-purpose breeds (i.e., tie-stall systems during wintertime and pasture access during summertime) and using fewer inputs [12,13]. When specialized high-yield dairy breeds are preferred, all the other farm characteristics and management practices tend to mimic intensive farming systems found in the lowlands [14].

The aim of this study was to investigate AMU in different dairy farming systems by comparing the treatment incidence in mountain farms with specialized high-yield dairy breeds or with dual-purpose breeds raised for milk production to the treatment incidence in lowland farms with specialized high-yield dairy breeds or with dual-purpose breeds raised for milk production.

## 2. Materials and Methods

### 2.1. Study Design and Data Collection

This study was carried out in Northeastern Italy from January 2016 to December 2017. The main factors tested were the farm location (Mountain vs. Lowland) and the breed reared (specialized high-yield dairy breed—Holstein—vs. dual-purpose breed raised for milk production—Simmental). Therefore, four groups of farms were identified: Mountain-Holstein (MH), Lowland-Holstein (LH), Mountain-Simmental (MS), and Lowland-Simmental (LS). Seven farms were assigned to each group based on the following criteria: farm location (above or below 600 m asl, defined as mountain or lowland, respectively), breed type (>70% of Holstein or Simmental per herd), and farmer’s willingness to be part of the study, thus representing a convenience sample. Further information about the herd was provided by farmers or retrieved from farm records and official test days. Data on treatment records were collected with a time unit of one year per farm and retrieved from the farms’ paper journal. In accordance with the Italian legislation in force, farmers had to keep records of the official identification number of the animal treated, animal species and sex, date of first and last administration, drug name, the reason for treatment, and meat and milk withdrawal periods. They were not obliged to record the dosage used. Instead, they had to keep track of the amount of residual drug at the end of the treatment.

### 2.2. Data Editing

Data collected were reported in an Excel spreadsheet. Each animal identification number was recorded as “one treatment” considering only antimicrobials (AM) for the aims of this study. Based on the daily paper records, it was not always possible to distinguish the age category of the animal treated (Cow vs. Heifer vs. Calf). Where possible, however, specific drugs were assigned to animal categories (i.e., Calf and Dry cow), based on the specific indications of the drug’s prescription label. All the other drugs were assigned to the generic Cow category. Drugs with specific indications for calves and drugs for oral administration only, or drugs not allowed to be administered to cows, were considered as calves’ treatments and were, therefore, excluded from the study. Spry AM for external use and ruminal preparations (monensin) were excluded too.

Each AM was searched in the official pharmaceutical handbook [15] by the commercial name indicated in the paper records and was classified by its active substances. The active substances were then grouped by main families (penicillins, cephalosporins, etc.), to which combination drugs were assigned based on the main substance, according to the WHO guidelines for Anatomical Therapeutic Chemical (ATC) classification [16], and finally assigned to the respective class of importance for human health as defined by the WHO ranking of CIA for human medicine [1]. “Lyncosamides and Spectinomycin” was the only main family of drugs composed by two AM, and it was assigned to the highest class of priority between the two AM (i.e., HIA). Antimicrobials were further classified by administration route: injectable, intramammary, and intrauterine.

### 2.3. Quantification of Antimicrobial Consumption

The antimicrobial usage of each farm was described by treatment incidence (TI) for each AM group and represented the number of treatments per 1000 cow-days at risk, calculated by the following formula:

$$\mathrm{Treatment}\;\mathrm{Incidence}\;(\mathrm{TI})\;=\;\frac{\mathrm{treatments}\;(n)}{\mathrm{cows}\;(n)\;\times \;\mathrm{observation}\;\mathrm{period}\;(\mathrm{days})}\;\times \;1000$$

Treatment incidence is considered the most accurate method to describe differences in drugs’ usage among systems at a high-resolution level [17] and was used in several studies investigating AMU [8,18]. Additionally, the time at risk of being treated allowed us to correct for missing data due to the fact that three farms provided access to farm records for a shorter time period (210, 108, and 270 days for a MH, MS, and LS farm, respectively), although the observation period was set at 365 days. For this reason, we decided to calculate the TI instead of the cumulative incidence. Each treatment was considered an independent event (i.e., the treated animal was considered immediately recovered), in which a full course dose was given to animals, as suggested by Menéndez González and colleagues [3].

### 2.4. Statistical Analysis

The median, minimum, and maximum of the TI were determined for each farm group, antimicrobial class, and administration route.

Due to the fact that TI was not normally distributed, differences among farm groups (MH, LH, MS, LS) were compared using the Kruskal–Wallis non-parametric test. Provided significance, post-hoc tests with Steel–Dwass–Critchlow–Fligner adjustment were applied to seek which of the pairwise comparisons were responsible for the overall difference. To avoid a high proportion of tied values, dependent variables for which the percentage of zero values was greater than 40 were excluded from the statistical analysis.

The analyses were performed by XL-Stat (version 19.1, Addinsoft 1995–2019) and the level of significance was set at *p* < 0.05.

## 3. Results

### 3.1. Farm Characteristics

Results of farms’ descriptive statistics are reported in Table 1. All farms were located in the Northeastern regions of Italy and, more precisely: MH farms were located in South Tyrol and Veneto, LH farms in Veneto, and MS and LS farms in Friuli Venezia Giulia. Farmers were all, on average, in their forties. Lowland farms were larger in herd size (≥75 adult cows on average), they all used free-stalls, and none allowed cows on pasture during summer. The MS group had the lowest number of cows reared per farm and the highest number of tie-stall barns, as well as the highest number of farms providing cows with pasture access during summer. The highest milk yield (expressed as mature equivalent) was recorded for LH, whereas the lowest was for MS farms. The latter, moreover, had the highest longevity (expressed as number of lactations per cow) and the most variable somatic cell count compared to all the other groups.

### 3.2. Treatment Incidences

**Figure 1:**
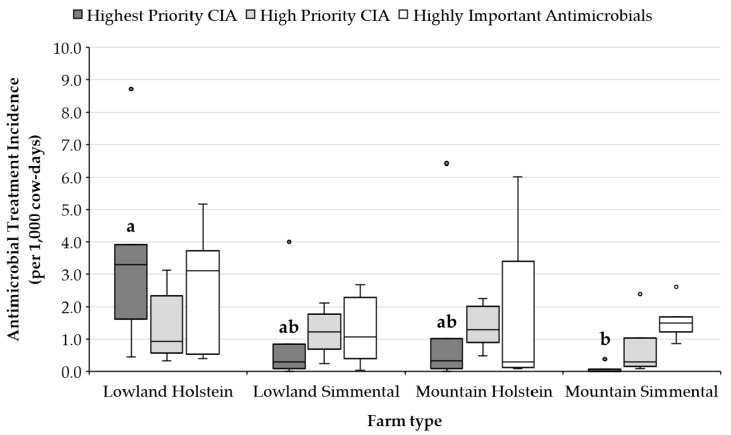
Antimicrobial treatment incidence (TI; treatments per 1,000 cows-days) by type of farm and WHO (World Health Organization) classification of critically important antimicrobials for human health (CIA) [1]. Median (horizontal lines), interquartile ranges (boxes), and 95th percentile (whiskers) are represented, and outliers are plotted separately as dots. Variables of the same color with different letters (a, b) differ (*p* < 0.05).

As shown in Figure 1, antimicrobial usage varied both within and across farming systems and breed types. The average total TI was 7.5, 4.1, 3.4, and 2.3 for LH, MH, LS, and MS respectively. Critically important antimicrobials, including both HP-CIA and H-CIA, accounted, on average, for about 60% in all farm types, with the exception of MS group, where CIA accounted for about 30% of all antimicrobial treatments. Significant differences among groups were found only between the overall TI, as well as HP-CIA TI, in LH and MS (*p* < 0.05).

**Figure 2:**
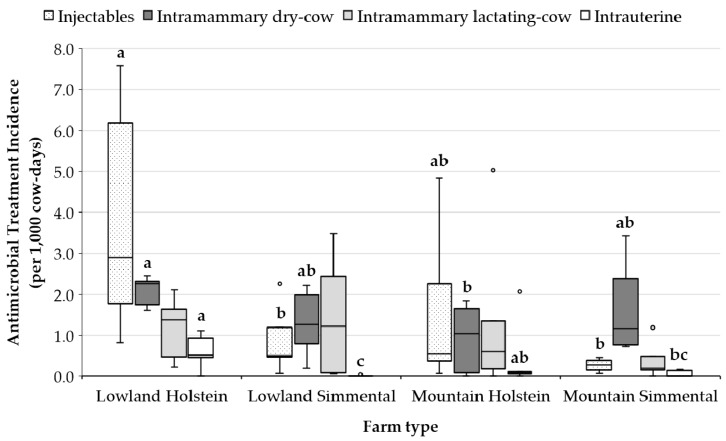
Antimicrobial treatment incidence (TI; treatments per 1000 cows-days) by route of administration and type of farm. Median (horizontal lines), interquartile ranges (boxes), and 95th percentile (whiskers) are represented, and outliers are plotted separately as dots. Variables of the same color with different letters (a, b) differ (*p* < 0.05).

In terms of administration route (Figure 2), the overall TI for systemically administered (i.e., injectable) antimicrobials was significantly higher in LH compared to LS and MS (*p* < 0.05). About intramammary AM, some differences among groups were found only for those drugs used for mastitis therapy, with a higher TI for LH farms, whereas the TI for dry cows was similar in all the groups. Significant differences were also highlighted in the overall TI for intrauterine therapy between LH and LS and between LH and MS, where LH had the highest value and LS the lowest (Figure 2).

When looking at antimicrobial classes for injectable drugs, TI for HP-CIA, such as third- and fourth-generation cephalosporins and fluoroquinolones, was significantly higher in LH only compared to MS (Table 2). When looking at intramammary treatments, a significant difference between TIs was found through post-hoc testing in LH and MS for first- and second-generation cephalosporins (HIA) used for mastitis therapy (Table 3). As intrauterine AMU was generally scarce in all farm groups and almost absent in some of them (e.g., LS), comparison by antimicrobial class was not carried out (Table 4).

## 4. Discussion

To our knowledge, this is the first study looking at differences in AMU across different dairy farming systems using data retrieved from official treatment records.

Treatment incidence was chosen as a unit given that other widespread metrics to quantify AMU, such as defined daily doses (DDDvet) or the garbage bin method, were deemed unsuitable for the purpose of this study. Unlike studies based on the amount of active substance consumption, in this case, the number of treatments was available and did not need to be estimated based on DDDvet and/or defined course doses (DCDvet) for animals [19]. These metrics are strongly influenced by the definition of the animal standard weight (425 kg for dairy cattle), prescribed dosage, treatment length, and average amount of active substance found in veterinary products available in nine European countries, excluding Italy [19]. As our study was investigating AMU in 28 Italian dairy farms with different breed types and body weights, the above-mentioned assumptions were going to introduce an unacceptable error in the study. On the other hand, a garbage bin method was not applicable in most small-scale farms, as the herd veterinarians were in charge of treatments’ administration by using their own drugs and, therefore, without leaving residuals, as well as empty vials, in the farms.

Despite the data used in this study were retrieved from treatment records validated by animal health officials, we could not always distinguish among age categories of the animals, as well as retrieve reasons for treatment. In regard to age categories, we chose to allocate to Cows all treatments that were not explicitly intended for calves, which might have led to a TI overestimation in adult dairy cows, especially in LH farms. For what concerns the reason for treatment, this information was not used, because it was often not specified, not readable, or too general to be classified.

In addition, it is worth noticing that the results are based on a convenience sample of farms rather than on a random sample, and extrapolations should thus be made with caution. The little number of farms investigated might not allow for robust statistical inference, but this study provided new knowledge on AMU in different dairy farming systems.

The results highlighted how AMU varied both within and across farming systems and breed types. Cephalosporins were the most frequently used antimicrobials, similar to what has been reported in previous studies [6,7,8]. The results of this work confirm the widespread use of systemically administered HP-CIA (i.e., 3^rd^ and 4^th^ cephalosporins) in specialized dairy farms represented by LH and, in a lesser extent, by MH. Reasons for this difference could lie in the correlation between milk production and AMU as being found in Canadian dairy farms [6]. Higher milk yield is known to be linked to metabolic disorders, mastitis, lameness, and other production diseases [20] for which prompt veterinary intervention is required.

Third- and fourth-generation cephalosporins are considered a veterinary critically important treatment option because of their efficacy, their short withdrawal period, and the presence of very few effective alternatives [21]. At the same time, third- and fourth-generation cephalosporines are also considered HP-CIA based on their importance to human medicine. The scope of the WHO characterization is to assist in managing antimicrobial resistance and in ensuring that antimicrobials are used prudently both in human and veterinary medicine [1]. Despite the fact that appropriate dosing is necessary at all times, strong limitations on the use of HP-CIA in veterinary medicine should also consider the consequences on cattle welfare. In fact, farmers and herd veterinarians might have to choose between treating a cow with a less effective substance having a longer withdrawal period or not treating the cow at all. For this reason, prudent AMU should go hand by hand with the introduction of disease prevention measures in order to ensure the maintenance of high welfare standards for the cows.

In contrast to AMU in LH, AM, in general, and HP-CIA, in particular, were seldom used in MS farms taking part in our study. Mountain farms have a generally smaller herd size than lowland farms [22], provide cows with access to pasture, and limit concentrates in the diet [23]. Lower production and traditional management practices could thus mitigate the production disease risk and the need for AM. The findings on the relationship between herd size, milk yield, and AMU also correspond to consumers’ perceptions of antimicrobial use in different farming systems [24].

Jones and colleagues [25] highlighted the role of consumers in inducing AMU changes on farms and suggested that the higher the proportion of income from milk production, the greater the likelihood of farmers exhibiting a positive intention to reduce AMU in response to public concern. All farmers in our sample were full-time farmers having milk production as their main source of revenue. The relatively young age of the farmers involved in this study could represent a further reason for showing a positive attitude toward consumer demands. In this regard, it is worth mentioning that consumers may not always appreciate the difference between prudent-use, misuse, overuse, and no-use of AM and their implications for animal health and welfare. For this reason, targeted health and welfare management plans including, for example, the introduction of selective dry-cow treatment in MS and the reduction of HP-CIA use in LH, should be carefully defined by the herd veterinarian and effectively communicated to consumers. This approach would help in the achievement of better animal health and welfare outcomes and the overall reduction of AMU while maintaining the support of properly informed consumers.

## 5. Conclusions

This study compared AMU in different dairy farming systems, characterized by their farm location (lowland vs. mountain) and breed type (Holstein vs. Simmental). The main differences were found in the overall TI of LH compared to MS, and particularly for what concerns HP-CIA, showing how mountain farms rearing dual-purpose breeds may represent a sustainable farming system in terms of AMU. The findings could be linked to several factors, such as lower milk production, smaller herd size, limited concentrate fraction in the diet, and pasture access provision. In an attempt to reduce AMU and mitigate AMR, farming practices such as those characterizing MS should be supported even beyond mountain areas.

## Figures and Tables

**Table 1:** Descriptive statistics (mean and SD) of farm characteristics for Lowland-Holstein farms (LH, *n* = 7), Lowland-Simmental farms (LS, *n* = 7), Mountain-Holstein farms (MH, *n* = 7), and Mountain-Simmental farms (MS, *n* = 7).

Characteristic ^1^	Farm Group			
	LH	LS	MH	MS
Farmers’ age	43 ± 11	44 ± 12	44 ± 5	42 ± 16
Farm altitude (mt asl)	133 ± 79	104 ± 93	873 ± 127	672 ± 218
Number of cows	112 ± 47	75 ± 14	46 ± 16	30 ± 10
Same breed type (%)	93.7 ± 10.7	96.7 ± 3.1	85.7 ± 14.0	96.9 ± 8.3
Cow housing (% of free-stalls)	100	100	86	14
Summer pasture (% of farms)	0	0	29	57
Mature equivalent milk yield (kg)	11,343 ± 1,743	8504 ± 656	9695 ± 874	5707 ± 1,052
Number of lactations	2.4 ± 0.2	2.5 ± 0.4	2.5 ± 0.2	3.7 ± 0.6
Somatic cell count (cells/mL × 1000)	210 ± 82	281 ± 92	203 ± 96	269 ± 110

^1^ For LH and MH: Percentage of Holstein cows, for LS and MS: Percentage of Simmental cows.

**Table 2:** Antimicrobial treatment incidence (TI; treatments per 1,000 cows-days) for injectable drugs used in Lowland-Holstein farms (LH, *n* = 7), Lowland-Simmental farms (LS, *n* = 7), Mountain-Holstein farms (MH, *n* = 7), and Mountain-Simmental farms (MS, *n* = 7).

Drug Class ^1^	Farm Type (Median, Min, and Max)				
	LH	LS	MH	MS	*p*-Value
HP-CIA					
Cephalosporins (3rd and 4th generation)	0.3 (0.0–2.7) ^a^	0.1 (0.0–0.7) ^a,b^	0.0 (0.0–1.0) ^a,b^	0.0 (0.0–0.2) ^b^	0.008
Fluoroquinolones	0.5 (0.2–2.1) ^a^	0.1 (0.0–0.5) ^a,b^	0.1 (0.0–1.2) ^a,b^	0.0 (0.0–0.2) ^b^	0.004
Macrolides	0.1 (0.0–1.6)	0.0 (0.0–0.4)	0.1 (0.0–1.7)	0.0 (0.0–0.0)	NA ^2^
H-CIA					
Penicillins	0.3 (0.0–2.3)	0.2 (0.0–0.4)	0.3 (0.0–1.3)	0.0 (0.0–0.3)	0.033
HIA					
Lyncosamides and Spectinomycin	0.1 (0.0–1.2)	0.0 (0.0–0.0)	0.0 (0.0–0.0)	0.0 (0.0–0.4)	NA
Sulfonamides	0.0 (0.0–0.3)	0.0 (0.0–0.2)	0.0 (0.0–0.6)	0.0 (0.0–0.2)	NA
Tetracyclines	0.2 (0.0–2.1)	0.0 (0.0–0.7)	0.1 (0.0–0.7)	0.0 (0.0–0.1)	NA

^1^ HP-CIA = highest-priority critically important antimicrobials; H-CIA = high-priority critically important antimicrobials; HIA = highly important antimicrobials. According to the World Health Organization (WHO) [1]. ^2^ NA = not applicable. The statistical test was not applicable, due to the high number of values equal to zero (>40%). ^a,b^ Medians within a row with different superscripts differ (*p* < 0.05).

**Table 3:** Antimicrobial treatment incidence (TI; treatments per 1,000 cows-days) for intramammary drugs used in Lowland-Holstein farms (LH, *n* = 7), Lowland-Simmental farms (LS, *n* = 7), Mountain-Holstein farms (MH, *n* = 7), and Mountain-Simmental farms (MS, *n* = 7).

Drug Class ^1^	Farm Type (Median, Min, and Max)				
	LH	LS	MH	MS	*p*-Value
Dry cows					
HP-CIA					
Cephalosporins (3rd and 4th generation)	1.9 (0.0–2.4)	0.0 (0.0–1.3)	0.0 (0.0–0.2)	0.0 (0.0–0.0)	NA ^2^
H-CIA					
Penicillins	0.0 (0.0–2.2)	0.0 (0.0–1.7)	0.4 (0.0–1.5)	0.0 (0.0–1.0)	NA
Rifamycins	0.0 (0.0–0.4)	0.0 (0.0–1.5)	0.0 (0.0–0.1)	0.0 (0.0–1.2)	NA
HIA					
Cephalosporins (1st and 2nd generation)	0.0 (0.0–0.8)	0.0 (0.0–2.0)	0.0 (0.0–1.5)	1.1 (0.0–2.4)	NA
Penicillins	0.0 (0.0–0.8)	0.0 (0.0–0.2)	0.0 (0.0–0.0)	0.0 (0.0–1.2)	NA
Lactating cows (mastitis therapy)					
HP-CIA					
Cephalosporins (3rd and 4th generation)	0.0 (0.0–0.2)	0.0 (0.0–1.6)	0.0 (0.0–2.4)	0.0 (0.0–0.0)	NA
H-CIA					
Penicillins	0.0 (0.0–0.8)	0.1 (0.0–1.7)	0.2 (0.0–1.3)	0.0 (0.0–1.2)	NA
HIA					
Amphenicols	0.0 (0.0–0.0)	0.0 (0.0–0.2)	0.0 (0.0–0.1)	0.0 (0.0–0.0)	NA
Cephalosporins (1st and 2nd generation)	0.9 (0.2–2.1) ^a^	0.1 (0.0–2.4) ^a,b^	0.0 (0.0–1.8) ^a,b^	0.0 (0.0–0.3) ^b^	0.034
Penicillins	0.0 (0.0–0.0)	0.0 (0.0–0.2)	0.0 (0.0–0.0)	0.0 (0.0–0.0)	NA
Sulfonamides	0.0 (0.0–0.0)	0.0 (0.0–0.1)	0.0 (0.0–0.0)	0.0 (0.0–0.0)	NA

^1^ HP-CIA = highest-priority critically important antimicrobials; H-CIA = high-priority critically important antimicrobials; HIA = highly important antimicrobials. According to the World Health Organization (WHO) [1]. ^2^ NA = not applicable. The statistical test was not applicable due to the high number of values equal to zero (>40%). ^a,b^ Medians within a row with different superscripts differ (*p* < 0.05).

**Table 4:** Antimicrobial treatment incidence (TI; treatments per 1,000 cows-days) for intrauterine drugs used in Lowland-Holstein farms (LH, *n* = 7), Lowland-Simmental farms (LS, *n* = 7), Mountain-Holstein farms (MH, *n* = 7), and Mountain-Simmental farms (MS, *n* = 7).

Drug Class ^1^	Farm Type (Median, Min, and Max)				
	LH	LS	MH	MS	*p*-Value
H-CIA					
Rifamycins	0.0 (0.0–0.7)	0.0 (0.0–0.0)	0.0 (0.0–0.0)	0.0 (0.0–0.2)	NA ^2^
HIA					
Cephalosporins (1st and 2nd generation)	0.0 (0.0–0.3)	0.0 (0.0–0.0)	0.0 (0.0–0.0)	0.0 (0.0–0.0)	NA
Tetracyclines	0.5 (0.0–0.9)	0.0 (0.0–0.0)	0.1 (0.0–2.0)	0.0 (0.0–0.1)	NA

^1^ HP-CIA = highest-priority critically important antimicrobials; H-CIA = high-priority critically important antimicrobials; HIA = highly important antimicrobials. According to the World Health Organization (WHO) [1]. ^2^ NA = not applicable. The statistical test was not applicable, due to the high number of values equal to zero (>40%). ^a,b^ Medians within a row with different superscripts differ (*p* < 0.05).
